# Silencing of ELK3 Induces S-M Phase Arrest and Apoptosis and Upregulates SERPINE1 Expression Reducing Migration in Prostate Cancer Cells

**DOI:** 10.1155/2020/2406159

**Published:** 2020-02-13

**Authors:** Yuanshen Mao, Wenfeng Li, Bao Hua, Xin Gu, Weixin Pan, Qi Chen, Bin Xu, Zhong Wang, Chao Lu

**Affiliations:** Department of Urology, Shanghai Ninth People's Hospital, Shanghai Jiao Tong University, School of Medicine, Shanghai 200011, China

## Abstract

ELK3, an ETS domain-containing transcription factor, participates in various physiological and pathological processes including cell proliferation, migration, angiogenesis, and malignant progression. However, the role of ELK3 in prostate cancer cells and its mechanism are not fully understood. The contribution of ELK3 to prostate cancer progression was investigated in the present study. We showed that silencing of ELK3 by siRNA in prostate cancer cell DU145 induced S-M phase arrest, promoted apoptosis, inhibited cell proliferation and migration *in vitro*, and suppressed xenograft growth in mice *in vivo*. In accordance with its ability to arrest cells in S-M phase, the expression of cyclin A and cyclin B was downregulated. In addition, the expression of p53 was upregulated following ELK3 knockdown, while that of antiapoptotic Bcl-2 was decreased. The migration inhibition may partly due to upregulation of SERPINE1 (a serine protease inhibitor) followed ELK3 knockdown. Consistently, downregulation of SERPINE1 resulted in a modest elimination of migration inhibition resulted from ELK3 knockdown. Furthermore, we found that the AKT signaling was activated in ELK3 knockdown cells, and treatment these cells with AKT inhibitor attenuated SERPINE1 expression induced by ELK3 silencing, suggesting that activation of AKT pathway may be one of the reasons for upregulation of SERPINE1 after ELK3 knockdown. In conclusion, modulation of ELK3 expression may control the progression of prostate cancer partly by regulating cell growth, apoptosis, and migration.

## 1. Introduction

Prostate cancer (PCa) is the fourth most common cancer type worldwide and the second commonly diagnosed cancer and the fifth leading cause of cancer-related death in males [1]. Although, the mortality rate of PCa has been reported to decline in many developed countries in recent years due to the early diagnosis because of improved screening and treatment, which is still increasing in some countries of Central and South America, Asia and Central and Eastern Europe [1–4]. Moreover, recent observations have shown that PCa is an increasingly serious disease among older adolescent and young adult males, possibly due to inadequate diagnosis, and may also be caused by obesity, lack of exercise, and environmental carcinogens [5]. However, the exact molecular mechanism of PCa progress is not yet fully understood. Therefore, better understanding of the molecular processes associated with PCa progress is needed to develop effective therapeutic strategies to treat PCa and prolong patient survival.

ELK3 (also called Net, SAP-2, or ERP) is an ETS domain-containing transcription factor that forms a ternary complex transcription factor (TCF) together with ELK-1 and serum response factor accessory protein-1 (SAP-1) and binds to a specific DNA sequence through a purine-rich GGA core sequence to regulate the expression of many genes including proto-oncogenes [6, 7]. Under basal conditions, ELK3 is a transcriptional repressor, which converts to a transcriptional activator in response to ratsarcoma viral oncogene homolog/extracellular regulated protein kinases (RAS/ERK) signaling and p38 mitogen-activated protein kinase (MAPK) pathway [8–10], and is involved in cell migration, angiogenesis, and malignant progression [11–14]. Several studies have showed that ELK3 is overexpressed in some cancer cells and correlates with cell migration and invasion [13–15]. Hypoxia-inducible factor-1*α* (HIF-1*α*) can regulate the genes related to cell proliferation, apoptosis, angiogenesis, and metastasis, playing an important role in cancer progression [16–18]. Lee et al. found that ELK3 expression was upregulated in CD133^+^/CD44^+^ liver cancer stem cells, and silencing of ELK3 attenuated their metastatic potential by regulating HIF-1*α* expression [12]. In breast cancer, suppression of ELK3 in MDA-MB-231 cells resulted in the loss of metastatic characteristics, mainly by activating GATA binding protein 3 (GATA3) to regulate the expression of cell-cell adhesion factors and tight junctional proteins [12, 19].

ELK3 is activated through phosphorylation of critical residues in the C-terminal domain [8–10]. The RAS-activated phosphorylated Net (P-Net) can stimulate vascular endothelial growth factor (VEGF) expression and promotes angiogenesis, both of them are coexpressed in some tumors such as PCa, Kaposi's sarcoma and head and neck cancer, and more striking was that p-Net was highly expressed in tumor cells but not in normal surrounding tissue [20]. Moreover, XRP44X, an inhibitor of RAR/ERK activity of ELK3, can inhibit the growth and metastasis of PCa cells *in vivo* in mouse model. The tumors from animals treated with XRP44X reduced the expression of ELK3 protein and genes containing ELK3-like binding motifs in their promoters, including some serine protease inhibitor members [14]. This study suggests that inhibition of ELK3 may also suppress the progression of PCa, but the underlying mechanisms are still remained unclear.

Serpin family E member 1 (SERPINE1), also called plasminogen activator inhibitor 1 (PAI-1), is a serine protease inhibitor that inhibits tissue-type plasminogen activator (tPA) and urokinase (uPA). Both tPA and uPA cleave plasminogen into plasmin, then plasmin combined with matrix metallopeptidases (MMPs) mediate the degradation of extracellular matrix (ECM), thus promoting invasion and metastasis [21]. Studies have demonstrated that SERPINE1 prevents invasion of cancer cells by inhibiting uPA protease activity [22]. Moreover, six transmembrane epithelial antigen of the prostate 2 (STEAP2) knockdown, accompanied by SERPINE1 upregulation, can reduce the invasive potential of PCa cells [23]. Silencing of deleted in liver cancer 1 protein (DLC1) upregulates PAI-1 expression and reduces migration in normal prostate cells [24]. These indicate that SERPINE1 may act as a downstream effector of some oncogenes, controlling the migration of PCa cells. More interestingly, Buchwalter et al. reported that homozygous mutant of ELK3 could increase the expression of PAI-1 and cause the migration defect of mouse embryonic fibroblasts [25]. So, whether ELK3 participates in the progress of PCa also partly by regulating the expression of SERPINE1? This study is performed to understand the roles of ELK3 in PCa cells and its mechanisms, and then provides a potential new dimension for better control of PCa.

## 2. Materials and Methods

### 2.1. Cell Culture and Reagents

PCa cell line DU145 was obtained from the Type Culture Collection of the Chinese Academy of Sciences (Shanghai, China), and maintained at 37°C under 5% CO_2_ in high glucose Dulbecco's modified Eagle's medium (Sangon Biotech, Shanghai, China) supplemented with 10% fetal bovine serum (Sangon Biotech).

Primary antibody for detecting ELK3 (PA5-68978) was bought from Invitrogen (CA, USA); those for SERPINE1 (13801-1-AP), Cyclin dependent kinase 2 (CDK2) (10122-1-AP), CDK4 (11026-1-AP), CDK6 (14052-1-AP), Cyclin D1 (26939-1-AP), Cyclin E1 (11554-1-AP), Cyclin A2 (18202-1-AP), Cyclin B1 (60186-1-Ig), p53 (10442-1-AP) and *β*-ACTIN (60008-1-Ig) were purchased from Proteintech (Wuhan Sanying, China). Antibodies for detection of Vimentin (3932), phospho-Akt (4060), Akt (4691) and horseradish peroxidase (HRP)-linked secondary antibodies (antirabbit IgG (7074P2) and antimouse IgG (7076P2)) were obtained from Cell Signaling Technology (Danvers, MA, USA). RIPA lysis buffer I (C500005) was purchased from Sangon Biotech (Shanghai, China).

### 2.2. RNA Interference

The small interfering RNA (siRNA) method was used to knock down the expression of ELK3 or SERPINE1. Two different siRNAs targeting ELK3 (ELK3-siRNA: 5′-GGAUCAGAAAC-AUGAGCAU-dTdT-3′ and 5′-GCACAGACACCAAAUGGAUdTdT-3′), SERPINE1-siRNA (5′-AAGCAGCUAUGGGAUUCAAdTdT-3′), and negative control siRNA (NC-siRNA: 5′-CUUACGCUGAGUACUUCGAdTdT-3′) were purchased from Guangzhou RiboBio Co., Ltd. (Guangzhou, China). The two ELK3-siRNAs were pooled together, and all siRNAs were used at a final concentration of 100 nM. Control cells were subjected to mock transfection with NC-siRNA. The cells were transfected using lipofectamine 2000 (Invitrogen, USA) according to the manufacturer's instructions.

### 2.3. Western Blot Analysis

Cells were lysed in RIPA lysis buffer and equal amounts of proteins were resolved with loading buffer. After thermal denaturation for 5 min at 95°C, the protein extracts were separated using 12% SDS-PAGE, and then transferred to PVDF membranes. The membranes were blocked in 5% skim milk-TBST for 1 h at room temperature and incubated with the indicated primary antibodies according to the manufacturer's protocol for overnight at 4°C and blotted with the corresponding secondary antibodies. The target proteins were visualized using an enhanced chemiluminescence reagent (Amersham Biosciences, Cardiff, UK) and imaged in the BioRad ChemiDoc™ XRS + System. The density of each band was measured using Image Lab™ software (Bio-Rad, Hercules, CA, USA). *β*-ACTIN was used as an internal control.

### 2.4. Cell Proliferation Assay

To determine the effect of ELK3 knockdown on PCa cell growth *in vitro*, DU145 cells were seeded into 96-well plates at 2500 cells/well, after incubated overnight cells were transfected with siRNAs, six parallel wells for each siRNA. Cell Counting Kit-8 (CK04, Dojindo Laboratories, Japan) was used to test cell viability daily for 4 days posttransfection. 10 *μ*L of CCK-8 was added to each well and incubated for 2 h at 37°C. The absorbance was read in a microplate reader (BioTek ELX800, BioTek Instrument Inc., USA) at 450 nm. Experiments were performed three times.

### 2.5. Cell Adhesion Assays

DU145 cells (2.5 × 10^5^) were seeded into each well of six-well plates. 72 h after transfection, cells were detached with 0.25% trypsin-EDTA, counted, and reseeded in 1% DMEM at 1 × 10^4^ cells/well on 96-well plate. The cells were incubated for 1 h at 37°C, rinsed in PBS, and the number of attached cells was counted under randomly selected five fields per well using microscope (magnification, ×100). The experiment was performed in triplicate.

### 2.6. Clonogenic Assay

24 h after transfection, cells were detached, counted, and reseeded in 10% DMEM at 100 cells/well on 24-well plate and cultured for 7 d. The cells were fixed with 4% paraformaldehyde, stained with crystal violet, and rinsed three times with PBS. After observed under microscope, the stained cells were dissolved in 33% v/v acetic acid (100 *μ*L per well) and the eluent optical density (OD) was measured at 570 nm in a microplate reader [26]. Experiments were performed in triplicate.

### 2.7. Cell Cycle and Apoptosis Analysis

After transfection with siRNA, cells were subsequently cultured for 72 h. For cell cycle analysis, cells were collected and fixed with 70% ethanol overnight at −20°C. After washed with cold PBS, the fixed cells were suspended in 500 *μ*L phosphate-buffered saline (PBS) containing ribonuclease A and stained with propidium iodide for 30 min in dark at room temperature. For apoptosis analysis, cells were collected and analyzed using the Annexin V-FITC apoptosis detection kit (BioVision, USA). All the cell suspensions were subjected to a FACSCalibur flow cytometer (BD Co., USA) to analyze the cell cycle or apoptosis.

### 2.8. Migration Assay

Cell migration assay was performed by using the migration chambers with 8 *μ*m porosity (Merck Life Science (Shanghai) Co., Ltd. Shanghai, China) according to the manufacturer's instructions. 5 × 10^4^ of ELK3 knockdown cells in 100 *μ*L of 1% FBS-DMEM were seeded into the upper chamber of the Transwell, and 600 *μ*L of 15% FBS-DMEM was added to the bottom chamber. After 16 h of incubation, the cells that had not migrated were removed using a cotton swab and that had migrated were fixed with 4% paraformaldehyde and stained with crystal violet. After observed under microscope, the stained cells were dissolved in 33% v/v acetic acid (100 *μ*L for each chamber) and the eluent OD was measured at 570 nm in a microplate reader. Experiments were performed in triplicate.

### 2.9. Mouse Xenograft Models

Nude mice (male BALB/c nu/nu, 4-week-old) were obtained from the Shanghai SLAC Laboratory Animal Co. Ltd., Chinese Academy of Sciences, and fed with standard laboratory mice diet and water ad libitium. Mice were maintained in accordance with the institutional guidelines for the care and use of laboratory animals.

To investigate the effect of ELK3 on DU145 cells growth in vivo, we generated xenograft subcutaneous tumors in nude mice according to the previous description with modifications [27]. 72 h after transfection, 2 × 10^6^ DU145 cells transfected with NC-siRNA or ELK3-siRNA in 100 *μ*L DMEM were subcutaneously injected into the right flank of mice, four mice in each group. 10 d after cells implantation, palpable tumors were formed, and then the tumor volume was monitored by measuring the length (*L*) and width (*W*) with calipers once every 3 d and calculated with the following formula: (*L* × *W*^2^) × 0.5. The experiments ended 25 d after tumor cell inoculation. Tumor weights were determined at the 25^th^ day, and the tumor growth curve was drawn.

### 2.10. Statistical Analysis

Data are expressed as mean ± SD. Student's *t*-test was used to assess between-group differences. Values of *P* < 0.05 were considered statistically significant (^*∗*^*P* < 0.05, ^*∗∗*^*P* < 0.01).

## 3. Results

### 3.1. Downregulation of ELK3 Inhibits DU145 Cell Proliferation, Adhesion, and Colony-Forming

**Figure 1:**
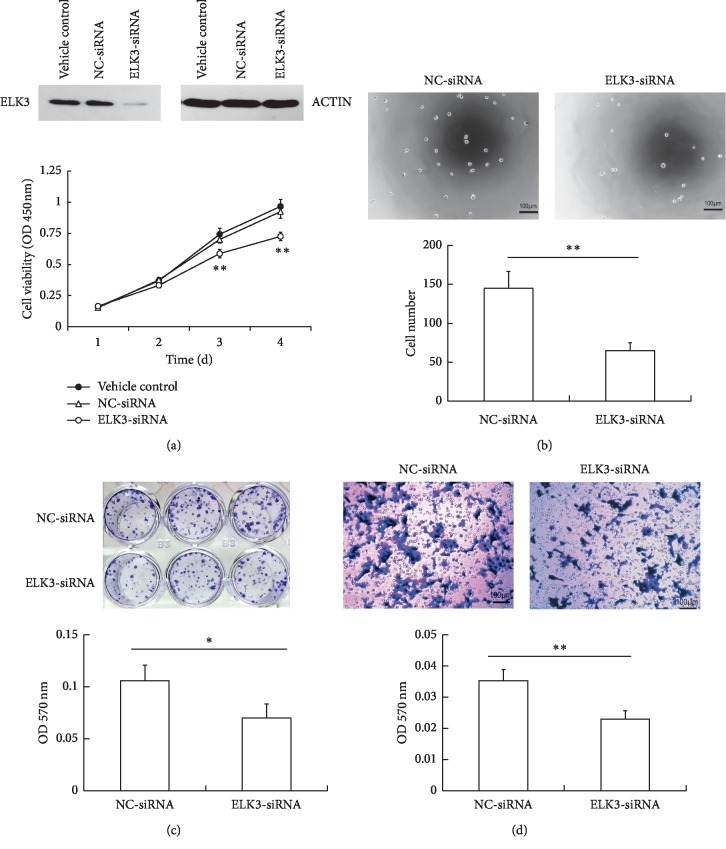
Silencing ELK3 inhibited the proliferation, adhesion, colony-forming, and migration of DU145 cells in vitro. (a) DU145 cells were transfected with siRNAs for 72 h, the expression of ELK3 and ACTIN were detected by western blotting (up panel); the effect of ELK3 knockdown on DU145 cell proliferation was examined by CCK-8 assay (lower panel). (b) Effect of ELK3 knockdown on cell adhesion. (c) Effect of ELK3 knockdown on cell colony-forming. (d) Transwell assay was performed to test the effect of ELK3 knockdown on cell migration ability. Cells were fixed with 4% paraformaldehyde and stained with crystal violet, then dissolved in 33% v/v acetic acid and measured the absorbance at 570 nm (c, d). Data were averaged from three parallel experiments and are given as mean ± SD. ^*∗*^*P* < 0.05, ^*∗∗*^*P* < 0.01, vs NC-siRNA.

Previous studies from other groups have shown that ELK3 upregulated in some cancer cells and associated with cell growth, migration, and invasion [13–15]. Here, we examined the effect of ELK3 on PCa cells *in vitro*. ELK3 was knocked down in DU145 cells by specific siRNA. The protein expression level of ELK3 evaluated by Western blot was significantly reduced in DU145 cells transfected with ELK3-siRNA compared with the one transfected with NC-siRNA. Then CCK-8 assay was conducted to determine the effect of ELK3 on cell proliferation. The growth of cells with ELK3 knockdown was significantly inhibited compared with that of controls (Figure 1(a)). Furthermore, the ability of cell adhesion and colony-forming was also obviously reduced by ELK3 silencing (Figures 1(b) and 1(c)). Transwell assay results revealed that the migration ability of ELK3-knocked down cells was lower than that of control cells (Figure 1(d)), suggesting that ELK3 may promote migration in DU145 cells. These results suggested that ELK3 would play a key role in PCa progression.

### 3.2. ELK3 Knockdown Results in S-M Phase Arrest and Promotes Cell Apoptosis

**Figure 2:**
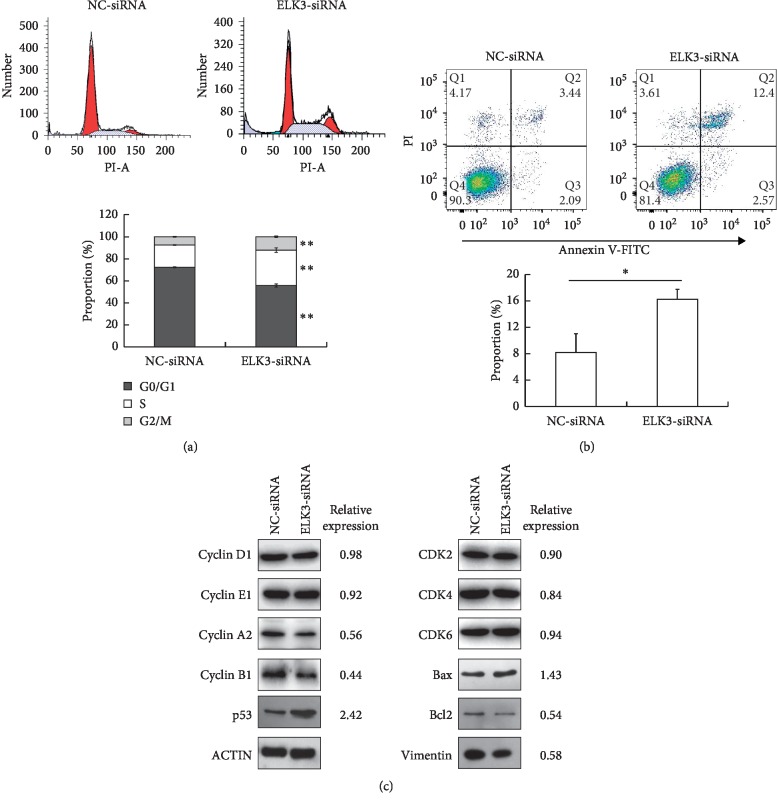
ELK3 knockdown results in S-M phase arrest and promotes cell apoptosis. (a) 72 h after transfection, the effect of ELK3 knockdown on cell cycle of DU145 cells was examined by flow cytometry. (b) Effect of ELK3 downregulation on apoptosis of DU145 cells. (c) Western blot analyzed the expression of cyclin D, cyclin E, cyclin A, cyclin B, CDK2, CDK4, and CDK6, p53, Bax, Bcl-2, and Vimentin. *β*-ACTIN was taken as the internal control, the expression of proteins was relative to NC-siRNA group. Data were averaged from three parallel experiments and are given as mean ± SD. ^*∗*^*P* < 0.05, ^*∗∗*^*P* < 0.01 vs NC-siRNA.

We further examined the effect of ELK3 knockdown on the cell cycle and apoptosis of DU145 cells using flow cytometry. The results showed that downregulation of ELK3 induced S-M phase arrest (Figure 2(a)) and promoted cell apoptosis (Figure 2(b)) in DU145 cells. Compared with the control group, ELK3 knockdown cells in G0/G1 phase decreased by about 23% and those in S phase and G2-M phase increased by more than 58% and 62%, respectively, and apoptotic cells doubled. In accordance with its ability to arrest cells in S-M phase, the expression of cyclin A and cyclin B was downregulated by ELK3 silencing, while that of cyclin D, cyclin E, CDK2, CDK4, and CDK6 did not had an obvious change (Figure 2(c)). The expression of p53 was upregulated following ELK3 knockdown, which of proapoptotic Bcl2-associated X protein (Bax) was just slightly increased, while that of antiapoptotic B-cell CLL/lymphoma 2 (Bcl-2) was decreased (Figure 2(c)). In addition, Vimentin (a marker of epithelial-mesenchymal transition, EMT) was also downregulated after ELK3 knockdown (Figure 2(c)). These results indicated that ELK3 exhibited an important function in the regulation of S-M phase cell cycle transition, cell apoptosis, and EMT.

### 3.3. ELK3 Silencing Inhibits the Growth of Xenograft Tumor *In Vivo*

**Figure 3:**
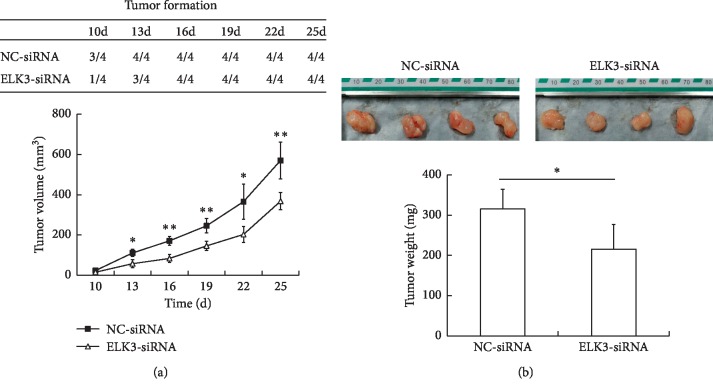
ELK3 knockdown inhibits the growth of xenograft tumor in vivo. DU145 cells transfected with NC-siRNA and ELK3-siRNA were injected subcutaneously into nude mice (4 mice for each group), respectively. (a) Tumor formation in the indicated time points (up table); tumor volume (lower graph). (b) Representative images of tumors formed in nude mice (up panel); tumor weight after sacrifice (lower panel). Data were averaged from 4 mice, and given as mean ± SD. ^*∗*^*P* < 0.05, ^*∗*^^*∗*^*P* < 0.01, vs NC-siRNA.

In order to study the effects of ELK3 knockdown on tumor growth *in vivo*, we generated xenograft subcutaneous tumors in nude mice. DU145 cells transfected with NC-siRNA and ELK3-siRNA were injected subcutaneously into nude mice, respectively. The palpable tumors were observed 10 d after cell implantation. ELK3 knockdown inhibited tumorigenesis: control cells formed tumor in three mice, while ELK3 knockdown cells only formed tumor in one mouse. All the control cells formed tumors on 13^th^ day, and all the ELK3 knockdown cells formed tumors on 16^th^ day. Tumor volume was estimated by measuring with calipers (Figure 3(a)) and by weighing the tumors after the mice were sacrificed (Figure 3(b)). ELK3 silencing inhibited the growth of primary tumors, as well as the final weight of the tumors at sacrifice. These results demonstrated that ELK3 is a critical modulator of the growth of xenograft tumors in nude mice.

### 3.4. ELK3 Knockdown Upregulates SERPINE1 and Inhibits Migration in DU145 Cells

**Figure 4:**
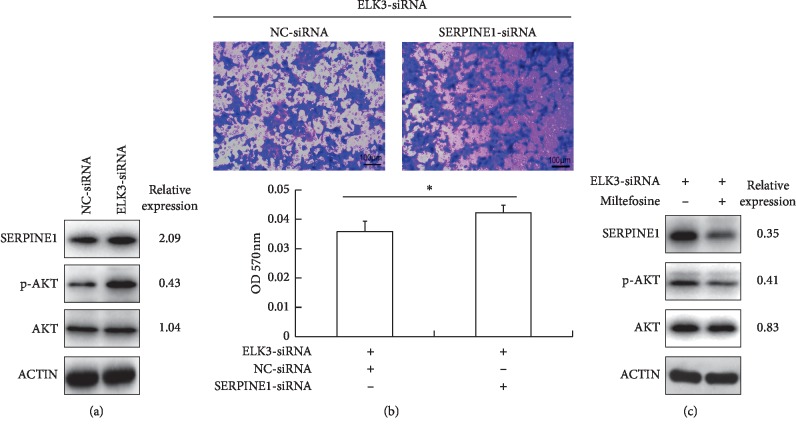
ELK3 knockdown inhibits migration of DU145 cells partly by upregulating SERPINE1 via AKT activation. (a) ELK3 knockdown resulted in upregulation of SERPINE1 and activation of AKT in DU145 cells. (b) Silencing SERPINE1 by specific siRNA in ELK3 knockdown DU145 cells modestly rescued the migration ability. (c) Inhibition of AKT with its inhibitor miltefosine (5 *μ*M) attenuated SERPINE1 expression induced by ELK3 silencing. Data were averaged from three parallel experiments, and given as mean ± SD. ^*∗*^*P* < 0.05, ^*∗∗*^*P* < 0.01, vs NC-siRNA.

SERPINE1 is a suppressor of cell migration, which is upregulated in the ELK3 knockdown cells in this study (Figure 4(a)). Previous studies from other groups showed that ELK3 promoted the migration of mouse embryonic fibroblasts through inhibition of SERPINE1 expression [25] and upregulation of SERPINE1 expression could reduce migration in normal prostate cells [24]. Thus, we examined whether SERPINE1 was the key factor in suppressing cell migration in ELK3 knockdown cells. By silencing SERPINE1 using specific siRNA in ELK3 knockdown DU145 cells, the migration defect was modestly rescued (Figure 4(b)).

### 3.5. Upregulation of SERPINE1 in ELK3 Knockdown Cells is Partly AKT Signaling Dependent

We found that the phosphorylation level of serine/threonine kinase (AKT) was significantly enhanced in ELK3 knockdown cells (Figure 4(a)). A previous study had also reported that phosphoinositide-3-kinase/Akt (PI3K/Akt) pathway was activated in ELK3 knockdown MDA-MB-231 cells [28], and this pathway is also known to promote SERPINE1 expression in other types of cells [29, 30]. So, we tested whether activation of AKT was required for SERPINE1 expression in our cell systems. 24 h after transfected with siRNA, the cells were treated with AKT inhibitor miltefosine (5 *μ*M) for another 48 h. Western blot showed that inhibition of AKT attenuated SERPINE1 expression induced by ELK3 silencing (Figure 4(c)), suggesting that activation of AKT pathway may be one of the reasons for upregulation of SERPINE1 after ELK3 knockdown.

## 4. Discussion

Our current study demonstrated that ELK3 acts as a positive regulator on PCa cell growth, migration, and EMT, and which knockdown results in cell migration inhibition may partly due to upregulation of SERPINE1 via the activation of AKT. ELK3 belongs to the ETS oncogene family and plays an important role in many biological processes such as cell proliferation, migration, invasion, and angiogenesis [11–14], which has been proved to promote the progression of several types of tumors including breast cancer, liver cancer, and squamous cell carcinomas [11–13, 15, 31]. On the contrary, some researchers reported that ELK3 was less expressed in cervical cancer and pancreatic cancer, and its ectopic expression inhibited the growth of these cancer cells [32–34]. These studies suggest that ELK3 expression in different types of tumors and cellular environment may lead to different functions.

ELK3 and the other two TCFs (ELK1 and SAP1) form complexes with serum response factor (SRF) to activate transcription when phosphorylated by MAP kinases. On the other hand, ELK3 has two inhibitory domains (NID and CID) usually leading to transcription repressing in the absence of SRF [8–10]. In PCa tissues, the expression of phosphorylated ELK3 was demonstrated to be higher in PCa cells than that in adjacent normal cells [20]. However, the role of ELK3 in PCa cells and its mechanism are not fully understood. In this study, we have found that knockdown of ELK3 significantly inhibited the growth of DU145 cells *in vitro* (Figure 1(a)) and *in vivo* (Figure 3), arrested cells in S-M phase, and promoted apoptosis (Figures 2(a) and 2(b)). In accordance with its ability to arrest cells in S-M phase, the expression of cyclin A and cyclin B was downregulated (Figure 2(c)).

A research from Sugimoto et al. showed that ELK3 mRNA was decreased when synchronized NIH3T3 cells in the S-M phase [35]. Wasylyk et al. identified a novel pyrazole XRP44X that inhibited fibroblast growth factor 2-induced ELK3 phosphorylation by the Ras-Erk signaling and led to G2-M cell cycle arrest in NIH3T3 fibroblasts and HUVEC endothelial cells [36]. Recently, Yoo et al. reported that knockdown of ELK3 suppressed cell proliferation with accumulation at the G1 cell cycle phase in MDA-MB-231 breast cancer cells [11]. However, in contrast, ELK3 over-expression inhibited the cell cycle progression of pancreatic carcinoma cells, resulting in an increase in G0/G1 phase fraction and a decrease in S phase fraction [34]. According to our findings and those of others, ELK3 may regulate cell cycle progression in a variety of ways, and they are consistent or different depending on cell type.

Furthermore, we demonstrated that knockdown of ELK3 inhibited migration of DU145 cells *in vitro* (Figure 1(d)), which may partly due to upregulation of SERPINE1 via activation of AKT (Figure 4). SERPINE1 is a suppressor of cell migration [21, 22] and its upregulation can reduce the invasive potential of PCa cells [23]. In this study, the silence of ELK3 increased SERPINE1 expression, indicating that ELK3 may play a negative role in the expression of SERPINE1 in PCa cells. ELK3 has been reported to inhibit SERPINE1 in mouse embryonic fibroblasts or NIH/3T3 cells by directly binding to a specific region of SERPINE1 promoter in the absence of SRF or in cell density-dependent manner [25, 37]. Therefore, knockdown of ELK3 may eliminate its inhibitory effect on SERPINE1 expression to some extent.

In addition, PI3K/AKT and MEK/ERK pathways have a dominant role in the expression of SERPINE1 in other cells [29, 30, 38]. A study on ELK3-associated signaling networks in breast cancer tissues revealed that expression of ELK3 was correlated with AKT activation [38]. The inhibitors of ERK1/2 (PD098059) and PI3K (LY294002) could attenuate ELK3 expression in endothelial cells induced by tumor necrosis factor α (TNF*α*) [30]. Interestingly, we found that the phosphorylation level of AKT was significantly enhanced in ELK3 knockdown cells (Figure 4(a)), which was similar to the result of ELK3 silencing in MDA-MB-231 cells reported by Park et al. [28]. Therefore, we tested whether activation of AKT was required for SERPINE1 expression in our cell systems. Treatment of the cells with AKT inhibitor miltefosine attenuated SERPINE1 expression induced by ELK3 silencing in DU145 cells (Figure 4(c)), suggesting that activation of AKT pathway may be one of the reasons for upregulation of SERPINE1 after ELK3 knockdown in our study.

**Figure 5:**
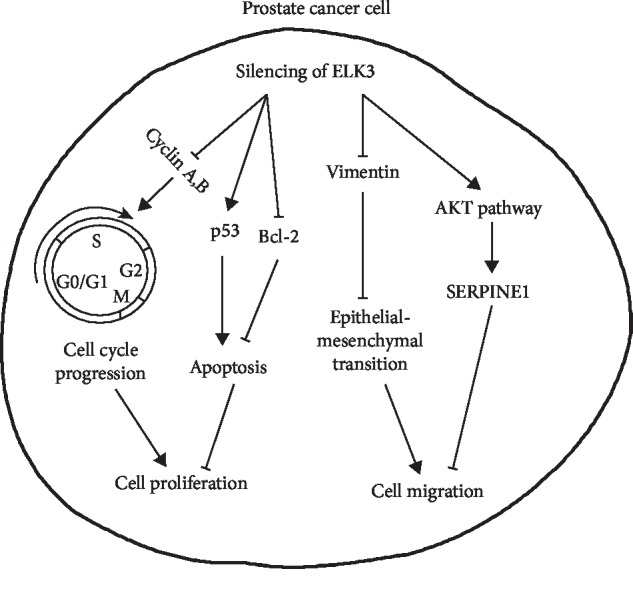
Schematic representation of the proposed mechanisms of ELK3 knockdown in prostate cancer cells. It showed that modulation of ELK3 expression may control the progression of prostate cancer partly by regulating cell growth, apoptosis, and migration.

Altogether, these findings indicate that ELK3 regulates cancer progression through different mechanisms of different cell types. Our current findings further enhance the regulatory effect of ELK3 on PCa cells. Silencing of ELK3 induced cell cycle arrest and apoptosis and reduced cell migration partly by upregulating SERPINE1 expression via activation of AKT signaling in PCa cells (a schematic representation of the proposed mechanisms is showed as Figure 5). Therefore, modulation of ELK3 expression may control the progression of PCa to a certain extent by regulating cell growth and migration, providing a new strategy for the treatment of PCa.

## Data Availability

The data used to support the findings of this study are included within the article.
